# Optical and Electrical Method Characterizing the Dynamic Behavior of the Fused Silica Cylindrical Resonator

**DOI:** 10.3390/s19132928

**Published:** 2019-07-02

**Authors:** Zhinan Qiu, Tianliang Qu, Yao Pan, Yonglei Jia, Zhenfang Fan, Kaiyong Yang, Jie Yuan, Hui Luo

**Affiliations:** College of Advanced Interdisciplinary Studies, National University of Defense Technology, Changsha 410073, China

**Keywords:** fused silica cylindrical resonant gyroscope, capacitive detection, frequency split, Q factor, laser Doppler vibrometer, LabVIEW program

## Abstract

Fused silica cylindrical resonant gyroscope (CRG) is a novel high-precision solid-wave gyroscope, whose performance is primarily determined by the cylindrical resonator’s frequency split and quality factor (Q factor). The laser Doppler vibrometer (LDV) is extensively used to measure the dynamic behavior of fused silica cylindrical resonators. An electrical method was proposed to characterize the dynamic behavior of the cylindrical resonator to enhance the measurement efficiency and decrease the equipment cost. With the data acquisition system and the designed signal analysis program based on LabVIEW software, the dynamic behavior of the fused silica cylindrical resonator can be analyzed automatically and quickly. We compared all the electrical measurement results with the optical detection by LDV, demonstrating that the fast Fourier transform (FFT) result of the resonant frequency measured by the electrical method was 0.12 Hz higher than that with the optical method. Thus, the frequency split measured by the electrical and optical methods was the same in 0.18 Hz, and the measurement of the Q factor was basically the same in 730,000. We conducted all measurements under the same operation condition, and the optical method was used as a reference, demonstrating that the electrical method could characterize the dynamic behavior of the fused silica cylindrical resonator and enhance the measurement efficiency.

## 1. Introduction

Solid-wave gyroscopes measure the angular velocity or angle by Coriolis effect [1,2,3]. The recognized hemispherical resonator gyroscope (HRG) is small in size, lightweight, extremely reliable, has prolonged service life, which plays an indisputable role in space applications. Currently, various types of solid-wave gyroscopes are now extensively used in aerospace, aviation, marine, and land application fields [4,5]. Cylindrical resonator gyroscope (CRG) with a fused silica cylindrical shell resonator is a novel solid-wave gyroscope, which comprises of a simple structure, a few key components, and is much easier to manufacture compared with HRG [6,7]. The cylindrical resonator is the core component, the modal parameters of which determine the signal-to-noise ratio, sensitivity and resolution, zero rate output, and bias stability of the gyroscope [8]. Efficient, precise, and reliable characterization of the dynamic behavior, including the resonant frequency, frequency split, and Q factor of cylindrical resonators, is imperative to assess and improve the design, manufacturing, trimming, and assembly process of cylindrical resonators. In particular, the trimming process of resonators needs real-time feedback of parameters to ascertain the tuning location and amount to attain efficient and high-precision frequency tuning. 

To date, various methods have been reported on the characterization of the dynamic behavior of resonators, including the optical method [9,10,11,12,13,14], piezoelectric method [15], acoustic method [16,17], and electromagnetic method [18]. Xi et al. illustrated an acoustic excitation and detection method for exciting and assessing the characteristics of a cylindrical resonator [16]. Panahi et al. proposed a noninvasive characterization method for microelectromechanical systems (MEMS) devices using aerodynamic forces [17]. However, such methods can only be operated under moderate vacuum level, where air damping creates a large loss in measuring the Q factor. Then, Pan et al. proposed an acoustic excitation and laser Doppler detection method to conduct the measurement [6,7], which was suitable for measuring the modal parameters of resonators made of brittle materials under moderate vacuum. Nevertheless, acoustic methods experience fundamental limitations in lower pressure situations, when the atmosphere starts to behave like particles. Wu et al. proposed a noncontact measurement system using electromagnetic excitation and microphone detection [18], which was simple in testing apparatus and with the easy-to-implement measurement; however, this system can only characterize metal resonators. He et al. proposed a laser self-mixing interferometry method for precision displacement measurement of the shell resonator gyroscope, which has high accuracy; however, this method was vulnerable to temperature variations [19]. Shoaib et al. proposed an optical measurement method to measure the dynamic behavior in MEMS containing a stroboscopic–interferometer system, a computer–microvision system, and laser Doppler vibrometer (LDV), realizing the remote control and measuring operation through internet access [20]. However, the implementation of these systems is complex and expensive, which are not suitable for the low-budget requirement.

Comparatively, Bu et al. investigated a measurement method based on the electrostatic excitation and capacitive detection (CD) for the MEMS gyroscope [21]. The resonator was excited by step excitation, and the structural parameters were determined using fast Fourier transform (FFT) and Morlet wavelet transform in one single fast measurement. For resonators with high Q factor and low-frequency split, excitation and detection must be determined in a high-vacuum environment and noncontact manner to decrease the effect of air damping on the measurement system [8]. Compared with the methods mentioned above, the electrostatic excitation and CD method offers advantages such as simple implementation, high precision, real-time feedback, high efficiency, and low cost.

This paper proposes an electrostatic excitation and CD method to characterize fused silica cylindrical resonators at 10^–3^ Pa under the designed vacuum chamber. The dynamic behavior of fused silica cylindrical resonators was analyzed automatically and quickly by a data acquisition and analyzing system using a data acquisition board and a LabVIEW program. We compared the results with those obtained through the widely used optical method based on an LDV. The designed vacuum chamber enables the simultaneous measurement of laser detection (LD) and CD. This paper is articulated as follows: First, a brief description of the resonator structure is provided; then, the vibration and detection principles of fused cylindrical resonators are provided, especially the electrical measurement technique is detailed. Hence, the results of the measurement of the resonant frequency, vibration amplitude, frequency split, and Q factor by the electrical and optical methods, respectively, are provided and compared in detail. Finally, the results of such measurements of the resonator are discussed, drawing a conclusion that this electrical method is a reliable method to characterize the modal parameters of the resonator before it is put into the product and integrated with the electronics, which decreases the testing cost and enhances the measuring sufficiency, especially for the high-precision frequency tuning process.

## 2. Basics

### 2.1. Structure of the Fused Silica Cylindrical Resonator

**Figure 1:**
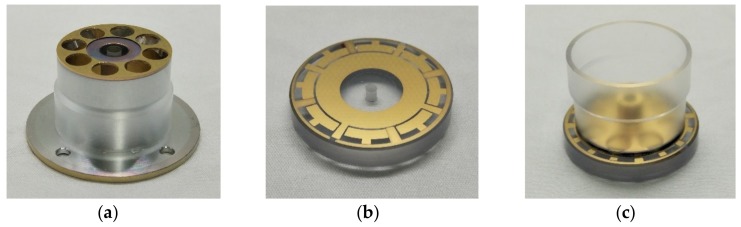
The structure of the fused silica cylindrical resonator: (**a**) The cylindrical resonator with a film coated on the bottom; (**b**) Plate electrode; and, (**c**) The integration of the cylindrical resonator and the plate electrode.

The specifics of the fused silica cylindrical resonator used in this study are detailed elsewhere [6]. The bottom plate of the resonator and the upper surface of the plate electrode are coated with Cr (~10 nm)–Au (~100 nm) film by magnetron sputtering. The resonator is excited and detected by the plate electrode. The metal film-coated resonator and electrode are vacuum-packaged as an integrated module by indium welding. After the integration, the gap is maintained at ~80 μm; therefore, no other step is needed to solve the problem of the assembling operation, which is highly convenient for experimental measurement and next assemble. Next, a glass-plate top sealing of the vacuum chamber was designed to enable LDV detection and, thus, compares different detection methods. Figure 1 shows the structure of the fused silica cylindrical resonator and the plate electrode. 

### 2.2. Vibration Principle 

**Figure 2:**
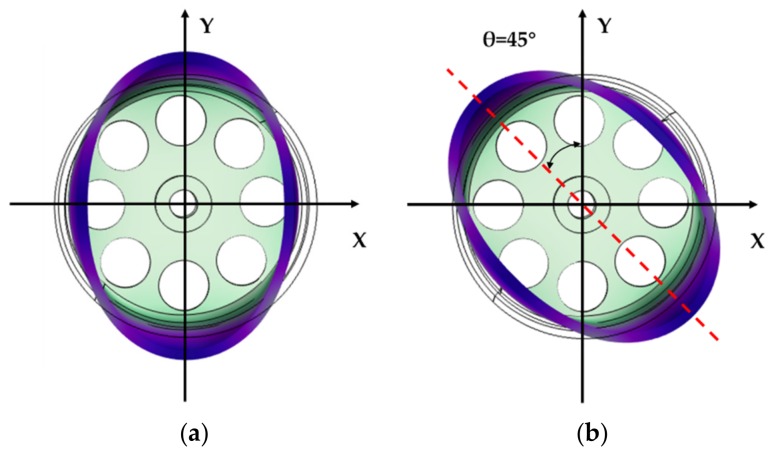
The *n = 2* vibration mode of the cylindrical resonator analyzed by *COMSOL Multiphysics 5.3a*: (**a**) The primary mode; and, (**b**) The secondary mode.

The cylindrical resonator is excited to vibrate at a *n = 2* mode, also called the *wineglass mode* comprising of 4 nodes and 4 antinodes [2], as is shown in Figure 2. In the force to rebalance (FTR) mode, the nodes and the antinodes maintained at the original position were named the 0° electrode axis by the electrostatic excitation, of which the vibration mode was called the primary mode. At an angular velocity input Ω, the secondary mode was induced along the 45° electrode axis, of which the amplitude of this mode was proportional to the magnitude of the input Ω. By assessing the variation of the amplitude of the secondary mode, we measured the angular velocity. The vibration equations of the resonator orthogonal decomposed along the 0°and the 45° electrode axes were as follows [22,23]:

(1) $$\begin{array}{c} \ddot{x}-2np\Omega \dot{y}+D_{xx}\dot{x}+D_{xy}\dot{y}+k_{xx}x+k_{xy}y=\frac{F_{x}}{m_{e}}\\ \ddot{y}-2np\Omega \dot{x}+D_{yx}\dot{x}+D_{yy}\dot{y}+k_{yx}x+k_{yy}y=\frac{F_{y}}{m_{e}} \end{array}$$

where *x* and *y* depict the displacement of the primary and secondary modes, respectively; *n* is the order of the vibration mode; *p* is the precession factor; *D_xx_* and *D_yy_* are damping coefficients of the resonator at 0° and 45° electrode axes, respectively; *D_xy_* and *D_yx_* are asymmetric damping of both axes, respectively; *k_xx_* and *k_yy_* are stiffness coefficients of both axes, respectively; *k_xy_* and *k_yx_* are asymmetric stiffness coefficients of both axes, respectively; *F_x_* and *F_y_* are forces applied on the electrodes at 0° and 45° electrode axes, respectively; and *m_e_* is the effective mass of the vibration region under electrostatic excitation.

Assuming the resonator is ideal, when no external angular velocity input exists, the obtained formula of the 0° electrode axis can be simplified as follows:

(2) $$\ddot{x}+D_{xx}\dot{x}+k_{xx}x=\frac{F_{x}}{m_{e}}$$

**Figure 3:**
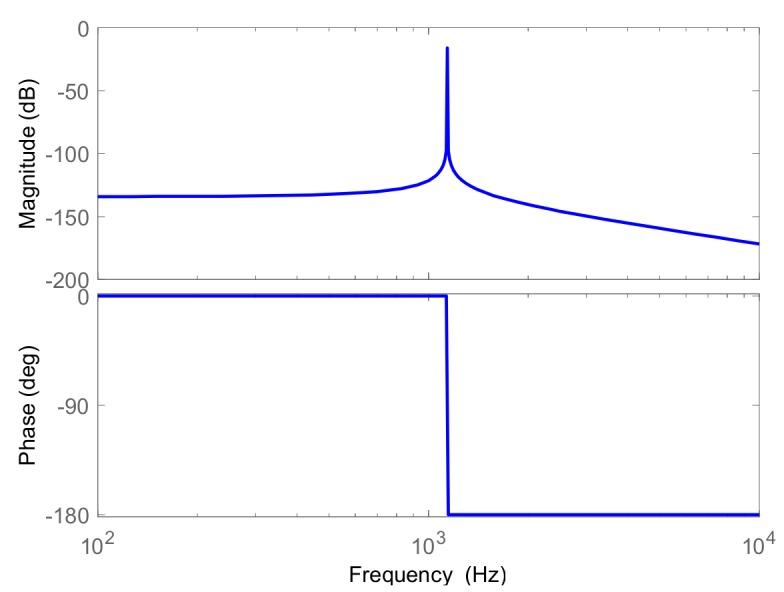
The amplitude frequency and phase frequency characteristic of the cylindrical resonator.

We used the Laplace transform of Equation (2) to obtain the transfer function from the excitation signal *F_x_* at the 0° electrode axis to the vibration displacement signal *x*, as the Laplace transform $G\left(s\right)$ can be expressed as follows:

(3) $$G\left(s\right)=\frac{F_{x}}{m_{e}\left(s^{2}+D_{xx}s+k_{xx}\right)}=\frac{F_{x}}{m_{e}\left[s^{2}+\left(\frac{\omega_{0}}{Q}\right)s+\omega_{0}{}^{2}\right]}$$

where, $\omega_{0}=\sqrt{k_{xx}}$ is the angular eigenfrequency of the CRG; $Q=\sqrt{k_{xx}}/D_{xx}$ is the quality factor of the resonator. As the specific parameters are brought into the Equation (3), we analyzed the amplitude frequency and phase frequency characteristic of the cylindrical resonator through Figure 3. The resonator itself could be considered as a bandpass filter, which only has response to its resonant frequency points. When the Q factor is higher, the passband is narrower, and the anti-interference ability is stronger. 

### 2.3. Detection Principle

**Figure 4:**
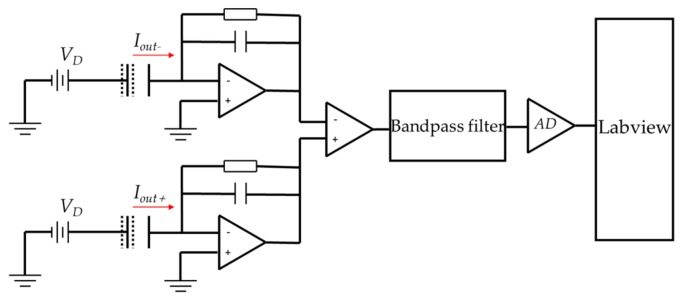
The schematic of the capacitive detection.

Figure 4 shows the simplified schematic of the CD model when the resonator was excited to vibrate, including the *C/V* converter, differential amplifier input, bandpass filter, analog to digital (AD) converter, and LabVIEW process program.

**Figure 5:**
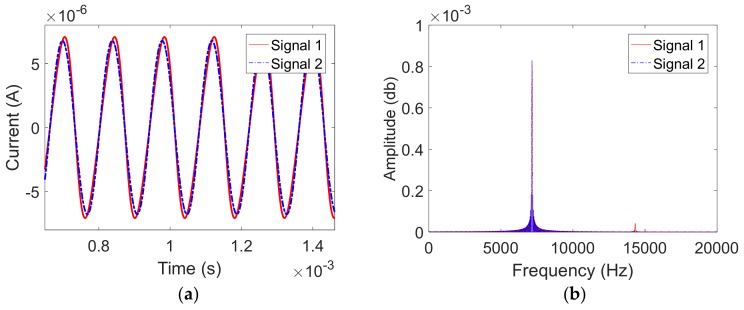
The weak current signal induced by vibration, where Signal 1 is the full form calculated by Equation (6) and Signal 2 is the first order approximation calculated by Equation (7): (**a**) The time domain of such Signal 1 and Signal 2; and, (**b**) The frequency domain of signal 1 and signal 2.

Based on the working principle of the capacitive gyroscope [24], a variation in the distance between the resonator itself and the plate electrode creates a variation in the capacitance, resulting in the variation of the weak sinusoidal current expressed as follows:

(4) $$i\left(t\right)=\frac{dQ}{dt}=\frac{dC\left(t\right)}{dt}V_{D}$$

where $C\left(t\right)=\frac{\varepsilon_{0}S}{x_{0}-x}$ is the real-time capacitance between the resonator and plate electrode, *V_D_* is the high DC voltage applied to the resonator, $\varepsilon_{0}$ is the vacuum permittivity. As the vibration of the cylindrical resonator can be expressed as follows:

(5) x=asinωt

where *a* is the vibration amplitude. Equation (5) is submitted into Equation (4) to obtain the specific expression of the current signal as expressed:

(6) $$i\left(t\right)=\frac{\varepsilon_{0}Sa\omega V_{D}}{{\left(x_{0}-asin\omega t\right)}^{2}}cos\omega t$$

where *S* is the effective area of the capacitance and *x_0_* is the initial distance between the plate electrode and the resonator. We made the Taylor expansion of the formula and kept the first order of the expression as follows:

(7) $$i\left(t\right)=V_{D}\frac{\varepsilon_{0}S}{x_{0}{}^{2}}a\omega cos\omega t=V_{D}\frac{C_{0}}{x_{0}}a\omega cos\omega t$$

where $C_{0}=\frac{\varepsilon_{0}S}{x_{0}}$ is the initial capacitance of the cylindrical resonator. Results calculated by Equations (6) and (7) were compared as shown in Figure 5a, where Signal 1 demonstrated the full form of the weak current signal *i(t)* calculated by Equation (6), while Signal 2 indicated the first order approximation of the weak current signal *i(t)* calculated by Equation (7). The FFT results of Signal 1 and Signal 2 were demonstrated in Figure 5b.

As shown in Figure 5a, results calculated by Equations (6) and (7) have approximately the same amplitude and frequency. Based on Figure 5b, both equations contain the resonant frequency component, while Signal 1 contains a higher-order frequency component more than Signal 2. By designing the bandpass filter appropriately, we could remove the higher-order frequency component and obtain the only resonant frequency component we wanted. Hence, the analysis of the vibration signal actually depends on the Equation (7). Thus, the measured signal is proportional to the high DC voltage *V_D_*, initial capacitance *C_0_*, vibration amplitude *a* and vibration angular frequency ω. However, it is inversely proportional to the initial gap *x_0_* between the plate electrode and the resonator. As *V_D_*, *C_0_* and *x_0_* are constant coefficients, the vibration amplitude and the resonant frequency of the fused cylindrical resonator can be known from the Equation (7).

## 3. Measurement Results

### 3.1. Frequency Measurement

**Figure 6:**
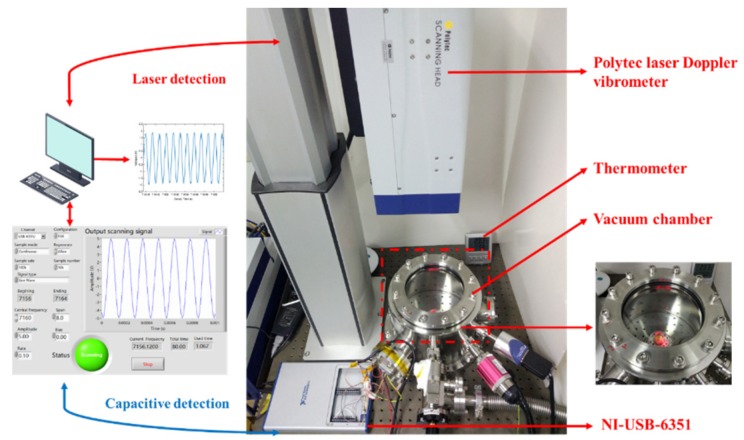
The testing present and apparatus of optical and electrical method characterizing the fused cylindrical resonator.

We excited the resonator by applying sinusoidal voltages through film electrodes on the plate, and the resonator itself was connected to high DC voltage. Both LD and CD were conducted simultaneously, as the equipment and testing present were shown in Figure 6. All excitation signals were generated by the data acquisition equipment NI-USB-6351 with the controlled program designed and operated in the LabVIEW software. In addition, the frequency measurement was measured by an automatically sweeping output program, by setting 7160 Hz as the central frequency, a span of 4 Hz, and sweeping time as 82 s, for which 82 s is the Polytec largest measuring time. All the relative parameters could be easily changed, as we used the LabVIEW program. The vibration information was recorded by LDV and the LabVIEW data acquisition module simultaneous, and FFT worked after all the data were obtained, respectively. 

**Figure 7:**
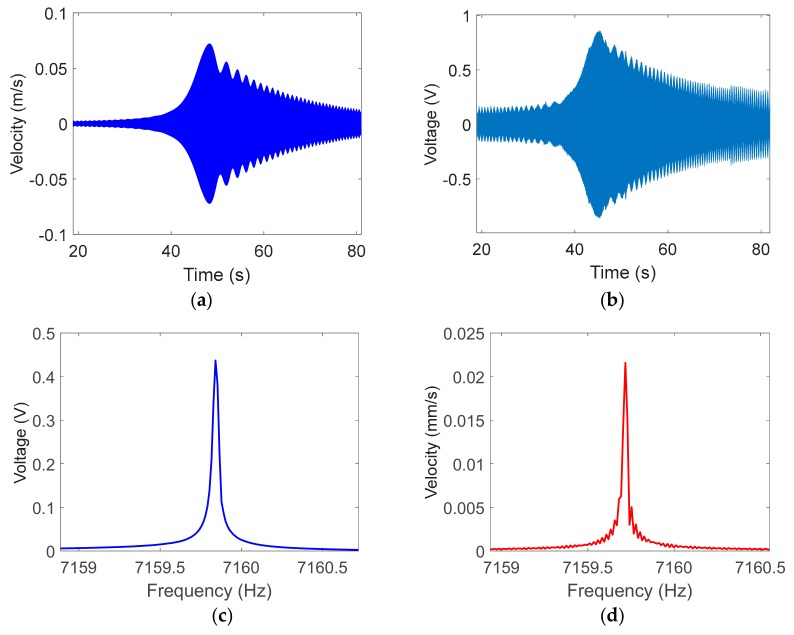
The results of frequency sweeping detected by laser Doppler vibrometer (LDV) and capacitive detection (CD): (**a**) The vibration velocity detected by LDV; (**b**) The vibration signal detected by CD; (**c**) Fast Fourier transform (FFT) results of laser Doppler detection; and, (**d**) FFT results of CD.

**Figure 8:**
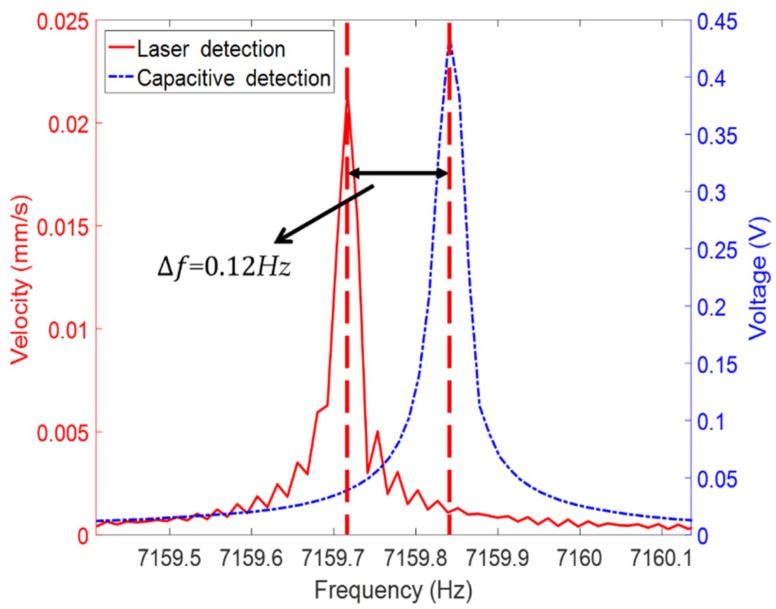
The resonant frequency measured by laser Doppler vibrometer (LDV; red) and capacitive detection (CD; blue).

First, the sweeping signal was applied to measure the resonant frequency of the resonator, according to Equation (3). As the frequency of the excitation signal approached the resonant frequency, the amplitude of the resonator kept increasing and, finally, reached to the maximum value at the resonance point. Then, as the frequency of the excitation signal deviated from the eigenfrequency, the vibration amplitude gradually decayed, as shown in Figure 7. The optical method measured the vibration velocity of the rim of the fused cylindrical resonator; the vibration velocity could reach the magnitude of 72 mm/s, as shown in Figure 7a. The electrical method measured the voltage converted from weak current through the plate electrode, the voltage could reach the magnitude of 0.85 V as shown in Figure 7b. Using the FFT program and the frequency spectrum of such two methods (Figure 7c,d), the difference is detailed in Figure 8.

Table 1 presents the frequency measurement data obtained. The resonant frequency was approximately 7159.8 Hz. The results revealed that the frequency measured by the optical method was smaller than that by the electrical method, and the difference was basically maintained at 0.12 Hz. The sample rate of both these methods was 20 kHz, and the resolution of the detection of the Polytec was 0.012 Hz. Notably, the frequency exhibited an increasing trend with each measurement, because the temperature of the lab increased from about 20.1 °C to 20.4 °C, as the resonant frequency positively correlates with temperature [25]; however, this was only the coarse measurement by the thermometer, which needs further study under the thermostat to determine the temperature characteristic of the filmed fused cylindrical resonator.

### 3.2. Measurement of the Vibration Amplitude

It is fundamental and vital to characterize the voltage–amplitude relationship to measure the vibration amplitude of the resonator precisely and quickly. We measured the vibration amplitude of the rim of the cylindrical along the 0° electrode axis and simultaneously conducted the LD and electrical detection. According to Equation (7), the vibration amplitude could be obtained through the amplitude of the current signal transferred into the voltage as we know the specific value of the precision resistor *R*. The equation is expressed as follows:

(8) $$U\left(t\right)=V_{D}\frac{C_{0}}{x_{0}}Ra\omega cos\omega t$$

**Figure 9:**
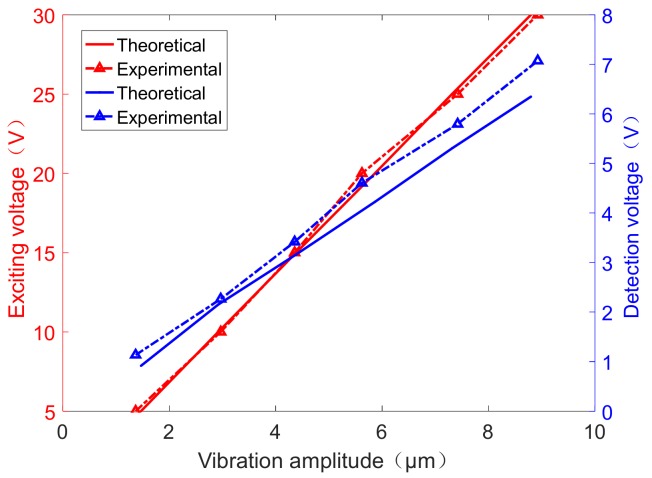
The relationship of the vibration amplitude between the exciting voltage (red) and the detection voltage (blue).

With the help of Equation (2), we calculated the vibration amplitude by the 4-order Runge–Kutta method in numerical solution theoretically. With the change of the different exciting voltages, we determined the detection voltage–vibration amplitude curve through Equation (8). Figure 9 presents the experimental and theoretical values.

We established a correlation between the exciting voltage and the vibration amplitude, shown with the red curve plotted with the solid line and dotted line in Figure 9, suggesting the theoretical and experimental values, respectively. Meanwhile, the correlation between the detection voltage and the vibration amplitude shown with the blue curve plotted with the solid line and dotted line implied the theoretical and experimental values, respectively. As the experimental results revealed a good match with the theoretical value, the standard deviation between the experimental results and the theoretical value was 0.22 V. The correlation between the vibration amplitude and the applying voltage can be expressed by fitting: y=0.293x−0.001; the linearity of the curve can be 0.981. Once the exciting voltage was determined, the vibration amplitude can be predicted. The correlation between the detection voltage and the vibration amplitude can be expressed by fitting: y=0.808x−0.0982; the linearity of the curve can be 0.997, and the standard deviation between the experimental results and the theoretical value was 0.14 V. Hence, the vibration amplitude of the resonator can be easily determined by these relationships within this range, which is crucial for investigating the further control theory of the fused cylindrical resonator. However, the accuracy of such characterizations warrants further improvement.

### 3.3. Measurement of the Frequency Split

**Figure 10:**
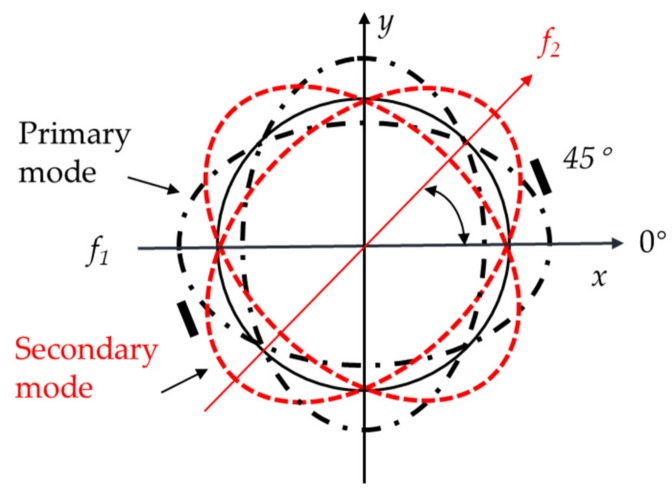
The resonator with two frequency axes,$f_{1}\neq f_{2}$, and a method to measure the frequency split.

For an idea cylindrical resonator, there is perfect symmetry along the circumference of the fused silica cylindrical resonator. In addition, the two eigenmodes along the two electrodes axes should have equal eigenfrequencies, and the axis of the eigenmode is fixed along the position of the excitation [26]. However, owing to various defects existing in the actual manufacturing process, leading to two inherent frequency axes make the eigenfrequencies unequal, $f_{1}\neq f_{2}$ (Figure 10). The imperfections lead to a small frequency split, simultaneously changing the orientation of the mode shape relative to the resonator [27,28,29,30]. Then, the standing waves vibrate along a higher-frequency axis or a low-frequency axis. Hence, there exists a deflection between the excitation position and the eigenmode. At the position of the excitation electrode in neither of the inherent frequency axes, the amplitude of the resonator in the φ direction can be expressed as follows [30,31]:

(9) $$Z\left(\varphi ,t\right)=Acos\left(2\varphi \right)\cos\left(2\varphi_{0}\right)\cos\left(2\pi f_{1}t\right)+Asin\left(2\varphi \right)\sin\left(2\varphi_{0}\right)\cos\left(2\pi f_{2}t\right)$$

where *A* is the amplitude of the vibration; φ is the angular position of the detection point; $\varphi_{0}$ is the direction of the wave relative to the higher-frequency axis; $f_{1}$ and $f_{2}$ are the frequency of the two axes, respectively.

**Figure 11:**
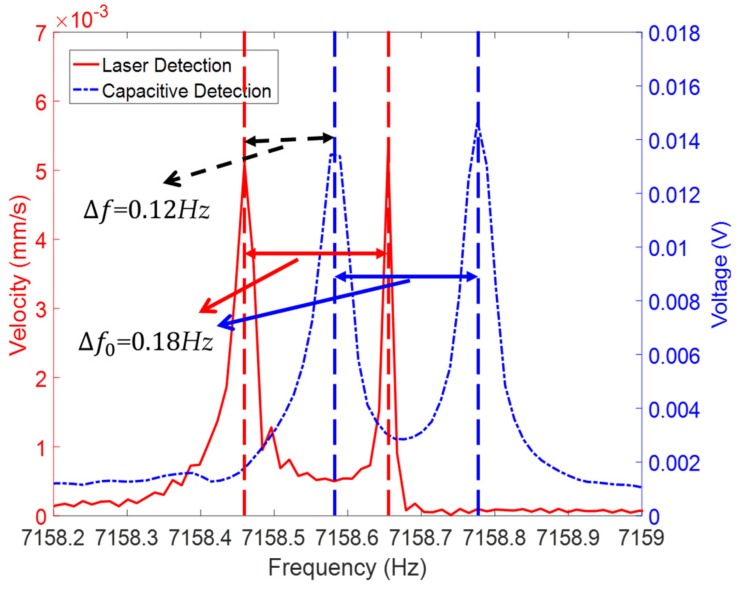
The frequency split measured by laser Doppler vibrometer (LDV; red) and capacitive detection (CD; blue).

When the resonator was not excited in any of these two eigen axes, usually performed at 22.5°, it locates in the mediate position between both eigen axes. Then, the vibration contains the information of two modes (Equation (9)). Thus, we could measure the vibration signal and analyze the frequency spectrum using FFT, obtaining the frequency split. Figure 11 shows the frequency split value measured by LDV and CD. 

Consequently, the frequencies of both modes measured by CD were 0.12 Hz larger than that measured by LDV, which corroborates the result of the frequency measurement discussed in Section 3.1. As shown in Figure 11, the frequency split of this resonator is 0.18 Hz, and the results of the optical and electrical methods are the same. The resolution of the FFT of the Polytec was 0.012 Hz; by increasing the amount of the acquired data through increasing the sweeping and measuring time *T*, the resolution could be enhanced as the sample rate is higher than two times of the measured signal, as the resolution of the FFT result can be expressed:

(10) $$R_{e}=\frac{1}{T}$$

The operating time of the electrical method can be set to >1000 s, of which the resolution of the FFT can be enhanced to 0.001 Hz, which is vital for the low-frequency split detection and the fundamental for the high-precision trimming operation.

### 3.4. Q Factor Measurement

The Q factor is a vital parameter for a gyroscope’s performance, representing the ratio of storage and loss in unit time. The higher the Q factor, the better the gyroscope performance [22]. Currently, two common methods to measure Q factor are −3dB bandwidth method and ring-down method [32]. The −3dB bandwidth method is calculated as Equation (11) under the FFT result of the frequency sweeping, where $f_{0}$ depicts the resonant frequency and $\Delta f_{r}$ represents the frequency −3dB bandwidth. Typically, the −3dB bandwidth method is used in the case of low Q factor, which can be quickly obtained by frequency sweeping and FFT. However, for the measurement with a high Q level, the ring-down method must be adopted [33]. When Q is >100,000, the resonant frequency is about 7 kHz, its bandwidth is only 10^−2^ Hz, requiring a very high accuracy of the measurement system.

(11) $$Q=\frac{f_{0}}{\Delta f_{r}}$$

**Figure 12:**
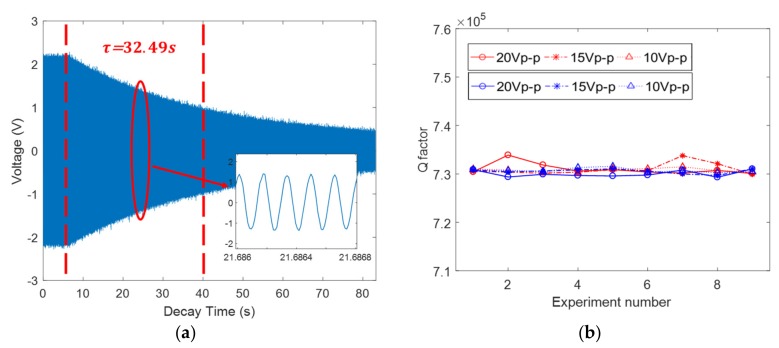
The Q factor measurement: (**a**) The decay time constant measured by capacitive detection (CD); and, (**b**) the Q factor measured by laser Doppler vibrometer (LDV; red) and CD (blue) in different excitation voltages.

The ring-down method measures the interval time of the amplitude from its normal vibration to its 1/e value, for which the calculated equation is provided as Equation (12). In our experiment, we used the ring-down method to measure the Q factor (Figure 12a).

(12) $$Q=\pi f_{0}\tau$$

where, τ is the decay time constant of the resonator.

We conducted a series experiment to measure the decay time constant and the Q factor of our resonator by the optical and electrical methods, respectively, in different excitation voltages. The results revealed that the decay time constant of this gyroscope is about 32.5 s; hence, the Q factor is approximately 730,000, calculated by Equation (12). In addition, the results established that the Q factor is not relevant to the amplitude of the excitation voltages from 10 Vp-p to 20 Vp-p, as shown in Figure 12b. The standard deviations of the Q factor measurement by LD and CD are approximately 0.71% and 0.69%, respectively, and the standard deviations between LD and CD are 0.73%–0.75% under the excitation voltages from 10 Vp-p to 20 Vp-p. Furthermore, the sample rate of CD was set to 100 KHz, for which we precisely obtained the natural oscillating waveform during the ring-down period, as shown in Figure 12a, which could be a method to measure its natural frequency.

## 4. Discussion

In this study, we compared the measurement results of the resonant frequency, vibration amplitude, frequency split, and Q factor of the film-coated fused silica cylindrical resonator with CD and LDV. The resonant frequency measured by CD is 0.12 Hz higher than LDV detection, with no difference in the setting parameter of the FFT algorithm between Polytec and the LabVIEW; however, the results presented a 0.12 Hz difference, which could be related to the analog-to-digital conversion or signal processing procedure in LDV. The resolution of the FFT directly determined the ability of the measurement of the frequency split. Theoretically, the resolution of the electrical method could obtain as high as 0.001 Hz by increasing the operation time as mentioned above, which is higher than that of the LDV and is crucial for the small-frequency split and the high Q factor measurement. Characterizing the modal parameters of the resonator is crucial to analyze the preliminary performance of a gyroscope, which could be used to examine the defective product and exclude the disqualification product timely. 

## 5. Conclusions

In this paper, a film-coated fused quartz cylindrical gyroscope and the designed plate electrode was introduced; the gyroscope was maintained at a high-vacuum level (<10^–3^ Pa) with the glass-plate top sealing of the vacuum chamber. The frequency of this cylindrical resonator is approximately 7160 Hz, the frequency split is 0.18 Hz, and the Q factor is 730,000; all these values are measured by optical and electrode methods, respectively, and CD exhibited a good accuracy in the measurement. Thus, CD provides a new scheme for the rapid and low-cost measurement, which has a universal application for such type of CRG or electrode pattern. Furthermore, the accuracy of CD determines the precision of the gyroscope and lays a good foundation for the realization of the closed control loop.

## Figures and Tables

**Table 1:** The resonant frequency measured by laser Doppler vibrometer (LDV) and capacitive detection (CD).

	$f_{T1}$	$f_{T2}$	$f_{T3}$	$f_{T4}$	$f_{T5}$	$f_{T6}$	$f_{T7}$
LDV	7159.717	7159.766	7159.79	7159.851	7159.875	7159.9	7159.961
CD	7159.840	7159.890	7159.914	7159.975	7160	7160.024	7160.087
Δf	0.123	0.124	0.125	0.124	0.125	0.125	0.126
